# Targeted immunotherapies for anaplastic lymphoma kinase-positive pediatric tumors: current advances and future perspectives

**DOI:** 10.3389/fimmu.2026.1912199

**Published:** 2026-08-14

**Authors:** Monica Maccagno, Roberta Tagliero, Roberto Chiarle, Claudia Voena

**Affiliations:** 1 Department of Molecular Biotechnology and Health Sciences, Molecular Biotechnology Center, University of Torino, Torino, Italy; 2 Department of Hematopathology, IEO European Institute of Oncology IRCCS, Milan, Italy; 3 Department of Pathology, Children’s Hospital and Harvard Medical School, Boston, MA, United States

**Keywords:** ALK-positive tumors, anaplastic lymphoma kinase (ALK), antibody-drug conjugate, CAR-T, immunotherapy, pediatric cancer, TCR-T, vaccine

## Abstract

Next-Generation Treatment for Pediatric Cancer: Advancing Immunotherapy through Combinations. Cancer remains one of the leading causes of disease-related mortality among children and adolescents. Despite advances in treatments, outcomes for many pediatric tumors remain limited, particularly in patients with high-risk, relapsed, or refractory disease. Standard therapies, including surgery, chemotherapy, radiotherapy, and stem cell transplantation, are frequently associated with severe long-term toxicities and secondary malignancies. Resistance and relapse remain major clinical challenges. Recent progress in cancer immunotherapy has revolutionized adult oncology, and remarkable clinical advances have been observed in several pediatric cancers. However, comparable benefits in most solid tumors remain limited. Among molecular targets, anaplastic lymphoma kinase (ALK) has emerged as a critical driver of tumorigenesis in several pediatric malignancies, including anaplastic large cell lymphoma (ALCL), neuroblastoma and inflammatory myofibroblastic tumor. While ALK tyrosine kinase inhibitors have demonstrated clinical benefit, the emergence of resistance highlights the need for alternative or complementary strategies. ALK can act as an oncoantigen, as shown by spontaneous ALK-specific humoral and T-cell responses in patients with ALK-positive ALCL, and ALK-directed immunotherapies have been developed and have shown efficacy in preclinical models of ALK-positive tumors, including ALCL and neuroblastoma. Therefore, ALK-directed immunotherapies represent biologically rational and promising approaches that may, if proven safe and effective in clinical trials, address resistance and improve long-term disease control. In this review, we will discuss ALK-specific immunotherapies, including ALK vaccines, ALK CAR-T and other cellular therapies, as well as ALK-targeting antibodies with a particular focus on pediatric ALK-positive tumors.

## 1 Introduction

**Figure 1:**
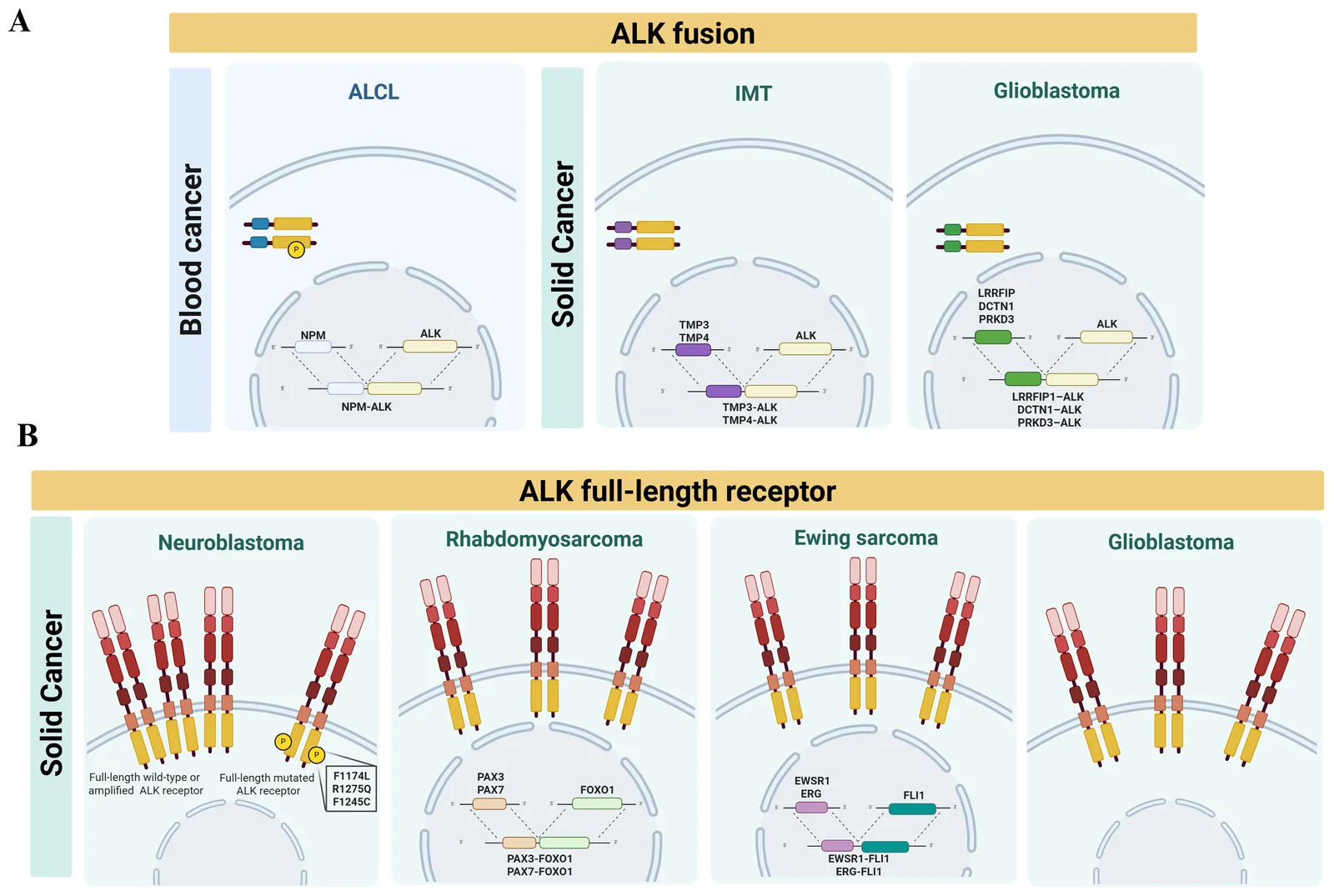
ALK in pediatric tumors. Pediatric cancers expressing ALK can be broadly categorized into those harboring ALK fusion proteins and those expressing the full-length ALK receptor. ALK fusion proteins are localized in the cytoplasm or nucleus, whereas wild-type, amplified, or mutated full-length ALK receptors are expressed at the cell membrane. **(A)** Pediatric cancers carrying ALK fusions. Among pediatric malignancies, ALK-positive anaplastic large-cell lymphoma (ALCL) is the only hematologic cancer commonly characterized by an ALK fusion. Specifically, the most common fusion partner of ALK in ALCL is nucleophosmin 1 (NPM1), resulting in *NPM-ALK* fusion. In contrast, ALK fusions in pediatric solid tumors are found in inflammatory myofibroblastic tumors (IMTs) and glioblastomas. IMTs harbor diverse ALK rearrangements in which the 3′ region of the *ALK* gene is fused to multiple partner genes, including *TPM3, TPM4, CLTC, CARS, SEC31L1, ATIC*. Among these, *TPM3–ALK* and *TPM4–ALK* are the most common and are depicted in the figure. Congenital and pediatric glioblastomas have also been reported to present *LRRFIP1–ALK, DCTN1–ALK*, and *PRKD3–ALK* rearrangements. **(B)** Pediatric cancers expressing full-length ALK receptor. Full-length ALK is expressed in several pediatric solid tumors, including neuroblastoma, rhabdomyosarcoma, Ewing sarcoma, and glioblastoma. Neuroblastoma may express wild-type, amplified, or mutated ALK, with the most frequent activating mutations being F1174L, R1275Q, and F1245C, which have been associated with resistance to ALK-targeted therapies. ALK-positive rhabdomyosarcomas are characterized by *PAX3–FOXO1* or *PAX7–FOXO1* fusions and surface expression of the full-length ALK receptor. Ewing sarcoma (EwS), typically driven by chromosomal translocations involving *EWSR1–FLI1* or *EWSR1–ERG*, can also express the wild-type ALK receptor, similar to glioblastoma.

Cancer remains a leading cause of death worldwide and is the most common cause of disease-related mortality in children and adolescents. Pediatric cancers account for approximately 1% of all tumors globally (1). Despite their relatively low incidence, pediatric tumors remain a significant clinical challenge, particularly due to their low mutational burden (2). The spectrum of cancers occurring in children is quite different from that observed in adults. Leukemia, central nervous system (CNS) tumors, lymphomas, and extracranial solid tumors, including neuroblastoma, bone and soft tissue sarcomas such as Ewing sarcoma and rhabdomyosarcoma, as well as Wilms tumor and retinoblastoma, represent the majority of pediatric malignancies (3). Standard therapy for pediatric childhood cancers still relies on surgery, radiation therapy, chemotherapy, and hematopoietic stem cell transplantation. Over the past several decades, combining these treatments has significantly improved long-term survival. However, increased survival rates have been accompanied by significant long-term toxicities, which affect quality of life, physical function, and overall health in childhood cancer survivors. Recently, targeted therapies and immunotherapy have been introduced for treating pediatric cancer, significantly increasing overall survival. Nevertheless, pediatric cancer outcomes for those who do not respond to treatment or experience relapse remain a challenge. In recent years, advances in genomic and epigenomic profiling have considerably improved the molecular characterization of pediatric cancers, enabling a more precise classification of tumor subtypes and the identification of oncogenic drivers involved in tumor initiation and progression. These discoveries have paved the way for the development of precision medicine approaches, including molecularly targeted therapies directed against specific genetic alterations. Among actionable targets in pediatric malignancies, anaplastic lymphoma kinase (ALK) has emerged as a particularly promising therapeutic candidate because its kinase activity is dysregulated by recurrent chromosomal rearrangements, point mutations, and gene amplifications. Indeed, ALK alterations have been described in several pediatric cancers, including anaplastic large cell lymphoma (ALCL), neuroblastoma (NB), inflammatory myofibroblastic tumor (IMT), glioblastoma (GBM) and rhabdomyosarcoma (RMS) (**Figure 1**) (4). This review focuses on the biological and clinical landscape of ALK-positive pediatric tumors, and on emerging ALK-directed immunotherapies, including ALK vaccines, ALK.CAR-T and other cellular therapies, ALK-targeting antibodies, and ALK T-cell engagers.

## 2 Anaplastic lymphoma kinase: structure, expression and oncogenic mechanisms

ALK is a receptor tyrosine kinase (RTK) belonging to the insulin receptor superfamily. Physiological ALK consists of an 18-amino-acid signal peptide, a long extracellular domain, a 21-amino-acid transmembrane segment, and an intracellular domain. The intracellular region includes a juxtamembrane segment, a tyrosine kinase domain, and a carboxy-terminal tail. The extracellular domain contains two MAM domains, one LDL domain, and a glycine-rich region, which are believed to play roles in protein interaction and cell adhesion (5). The activation of endogenous ALK requires ligand‐dependent receptor dimerization and autophosphorylation. During embryogenesis, ALK is highly expressed, and its signaling regulates neuronal development in the CNS, spinal cord, and enteric nervous system. After development, ALK is largely downregulated and remains only weakly detectable in some normal tissues (6). In cancer, ALK expression and activity are frequently reactivated through several genetic and molecular mechanisms, including chromosomal rearrangements, point mutations, gene amplifications. These alterations result in aberrant tyrosine kinase activation and trigger oncogenic downstream signaling mediated by MAPK, PI3K/AKT, and JAK–STAT pathways (7). Chromosomal rearrangements generate ALK fusion proteins in which constitutive kinase activation is typically driven by the dimerization mediated by the fusion partner. Activating point mutations and ALK overexpression, respectively, induce conformational changes and spontaneous receptor dimerization, leading to ligand-independent signaling. Overall, aberrant ALK kinase activity leads to hyperactivation and dysregulation of downstream pathways, ultimately promoting cellular transformation and cancer development. ALK fusion proteins have been identified in several pediatric tumors, including ALCL, IMT and rare cases of RMS, renal cell carcinoma (RCC) and pediatric papillary thyroid cancer. ALK point mutations and gene amplifications are commonly identified in NB. Amplifications or copy number gains of the *ALK* locus have also been reported in a subset of rhabdomyosarcoma cases (8). In some ALCL cases, secondary ALK mutations can arise in *ALK* fusion genes as a result of resistance to ALK tyrosine kinase inhibitors (TKIs). Notably, full-length wild-type ALK protein has also been detected in other pediatric solid tumors in which ALK is not the primary oncogenic driver of transformation, such as RMS, Ewing Sarcoma (EwS), GBM, pancreatoblastoma and Wilms’ tumor (8). In these contexts, ALK may still contribute to the specific tumor cell phenotype, thereby providing a tumor-associated antigen that can be exploited by ALK-specific immunotherapies (6).

## 3 Pediatric ALK-positive tumors and current therapeutic strategies

### 3.1 Anaplastic large cell lymphoma

Anaplastic Large Cell lymphoma (ALCL) accounts for 10%–15% of childhood and adolescent non-Hodgkin lymphomas (NHL). ALCLs are classified as mature T-Cell Lymphomas characterized by pleomorphic tumor cells with uniform strong expression of the CD30 receptor, also known as a member of the tumor necrosis factor receptor superfamily 8 (TNFRSF8) (9, 10). More than 85% of pediatric ALCL cases are ALK-positive due to the t (2, 5)(p23;q35) translocation, which generates the nucleophosmin (NPM1)–ALK fusion protein, the main oncogenic driver of these tumors (**Figure 1A**) (11, 12). Dimerization of NPM1-ALK via the N-terminal NPM1 domain leads to autophosphorylation and constitutive activation of the C-terminal ALK kinase domain. As a result, the activation of downstream signaling pathways, including PI3K/AKT/mTOR, RAS/ERK, JAK/STAT3 and STAT5, and RHO GTPase signaling pathway, enhances cell survival, inhibits apoptosis, promotes cell cycle progression, and tumor dissemination (7, 13).

The standard frontline treatment for pediatric ALCL is intensive multi-agent chemotherapy. Using this regimen, most patients achieve complete remission (CR); however, approximately 5% experience early disease progression during chemotherapy, and about 25% relapse, typically within the first months after treatment completion (14). Outcomes are particularly poor in patients with early relapse, refractory disease, or persistent minimal disseminated disease (MDD) and minimal residual disease (MRD).

In the relapsed or refractory setting, targeted therapies have emerged as important treatment options. Brentuximab-vedotin (BV), a CD30-directed antibody–drug conjugate, has shown CR rates of 41% to 66% in pediatric patients with ALCL when used as monotherapy (15, 16). BV is an anti-CD30 monoclonal antibody conjugated to the antimitotic agent monomethyl auristatin E (MMAE) through a cleavable linker; after binding to CD30-positive cells and internalization, MMAE is released and inhibits tubulin polymerization, leading to apoptosis of tumor cells (17). In addition, released MMAE can also induce “bystander killing” of adjacent tumor cells (18). The BV arm of the ANHL12P1 trial (NCT01979536) evaluated frontline BV added to alternating chemotherapy in newly diagnosed children and adolescents with ALK-positive CD30-positive ALCL. BV was well tolerated, with 2-yr EFS 79.1% and OS 97.0% (19). In addition, CD30-targeted CAR-T cell therapy has shown preliminary efficacy in patients with ALCL. Early clinical studies (NCT01316146) reported promising, although variable, responses, including complete and prolonged partial remissions, supporting further investigation and optimization of this therapeutic strategy (20–22).

Targeted therapies based on ALK inhibition with ALK TKIs have also been used to treat recurrent or refractory ALK-positive ALCL. Among these, crizotinib, a first-generation TKI, has shown significant clinical efficacy. In pediatric patients with recurrent or refractory ALK-positive tumors, remarkable responses were observed in children with ALK-positive ALCL, with complete response rates exceeding 80% when crizotinib was administered as a single agent in the phase I/II ADVL0912 trial (NCT00939770) (23). In the ANHL12P1 trial, the arm testing crizotinib combined with chemotherapy showed outcomes and survival similar to those of the BV and chemotherapy arm (19). Next-generation ALK TKIs, including alectinib, ceritinib, brigatinib, and lorlatinib, exhibit greater potency than crizotinib and offer the additional advantage of CNS penetration. In an open-label phase II trial in Japan, alectinib demonstrated an overall response rate (ORR) of 80%, leading to its approval for the treatment of patients with relapsed or refractory ALK-positive ALCL in 2020 (24). Furthermore, clinical trials evaluating the safety and efficacy of ceritinib (NCT01742286) and brigatinib (Briga-PED trial - NCT04925609) are currently ongoing in patients with relapsed or refractory ALK-positive ALCL. Ceritinib demonstrated an ORR of 75% in a cohort of 12 patients, while preliminary results from brigatinib trials have also shown promising safety and efficacy profiles (25, 26). The activity of the third-generation ALK inhibitor lorlatinib has not yet been evaluated in children with ALK+ ALCL; however, a clinical trial in previously treated adult patients is currently recruiting in Italy (NCT03505554).

Patients with ALK-positive ALCL mount a spontaneous humoral and cellular immune response against ALK, with high anti-ALK antibody titers and detectable CD8+ and CD4+ T-cell responses in peripheral blood. Taken together, the strong ALK dependency and the spontaneous immune response make ALK+ ALCL very attractive for ALK-directed immunotherapies, specifically as consolidation treatment after chemotherapy or ALK-TKIs, with the goal of eradicating the MRD and reducing the risk of relapse.

### 3.2 Neuroblastoma

Neuroblastoma (NB) is a pediatric cancer characterized by highly diverse clinical behavior, ranging from spontaneous regression to aggressive metastatic disease. NB is the most common extracranial solid tumor in children, accounting for approximately 10% of all pediatric cancer cases and about 15% of childhood cancer deaths (27). The disease derives from neural crest–derived cells of the sympathetic nervous system, most commonly originating in the adrenal medulla or along the sympathetic chain, including thoracic, abdominal, or cervical paraspinal ganglia. NB primarily affects very young children, with nearly 90% of cases diagnosed before the age of five and a median age at diagnosis of around two years (28, 29).

A key characteristic of neuroblastoma is its remarkable biological and clinical heterogeneity. NB is divided between low/intermediate neuroblastoma and high-risk neuroblastoma (HRNB). Low/intermediate-tumors appear as a localized disease that can spontaneously regress or be successfully treated with cytoreductive surgery and low-intensity chemotherapy. In contrast, HRNB often presents widespread metastases and remains difficult to treat, with survival rates below 50% despite intensive multimodal therapy that includes high-dose chemotherapy, surgery, radiotherapy, autologous stem cell transplantation, and anti-GD2 immunotherapy (28, 29).

Most NB cases occur sporadically; however, germline genetic alterations can increase an individual’s susceptibility to the disease. Familial NB accounts for approximately 2–3% of all cases and is frequently associated with highly penetrant germline activating mutations in the ALK gene, which are present in about 50% of familial cases (30). Less frequently, familial NB is linked to inactivating mutations in PHOX2B, a gene that encodes the transcription factor paired mesoderm homeobox protein 2B (31, 32). Genomic and chromosomal abnormalities are highly frequent, occurring in around 90% of both sporadic and familial cases (33, 34). A well-established driver of HRNB is MYCN amplification, located on chromosome 2p24, which represents a major prognostic marker linked to aggressive disease and poor clinical outcomes (35, 36). In addition to MYCN overexpression, proto-oncogene amplifications such as CDK4 and MDM2 and aberrant activation of RAS–MAPK signaling, together with p53 pathway inactivation, further contribute to tumor progression and treatment resistance (37, 38).

ALK alterations are among the most relevant oncogenic events in NB. In sporadic NBs, somatic ALK mutations are detected in approximately 6–12% of cases across all risk groups, whereas ALK amplification occurs in about 4% of patients with high-risk disease and is associated with poor prognosis. The most common activating ALK mutations (F1174, R1275, and F1245) are typically located within the kinase domain of the protein and can activate the ALK receptor in the absence of the ligand (**Figure 1B**) (39). Notably, ALK alterations, either mutations or amplification, often co-occur with MYCN amplification, suggesting a cooperative role in NB development, as demonstrated in zebrafish and murine models (40).

Given the aggressive nature of HRNB, several drugs have been evaluated, including ALK-TKIs. Crizotinib was evaluated in a phase I/II trial in pediatric patients with recurrent or refractory solid tumors (ADVL0912), where tumor responses were observed in 3 of 11 patients with ALK-mutated NB (23). However, prolonged treatment with crizotinib and other TKIs is often limited by the emergence of drug resistance. In NB, hotspot mutations such as F1174L and F1245C are associated with reduced sensitivity to crizotinib and can confer intrinsic resistance. Combining crizotinib with chemotherapy may help overcome this resistance through synergistic effects (41). Lorlatinib has also been evaluated in children and adults with relapsed and refractory ALK-driven NB, demonstrating anti-tumor activity with minimal toxicity (NCT03107988), and is now being investigated in the upfront setting for ALK-aberrant disease, replacing crizotinib in the NCT03126916 trial for HRNB (42).

NB cells are also characterized by high expression of another important and targetable surface molecular target, the glycolipid disialoganglioside (GD2) (43). Although GD2 is also present in some normal tissues, its expression is largely restricted to the CNS, peripheral nerves, melanocytes, and mesenchymal stem cells, making it an attractive target for immunotherapeutic strategies. Monoclonal antibodies targeting GD2, such as naxitamab and dinutuximab, are the first antigen-specific immunotherapies approved for the treatment of HRNB and have improved outcomes when combined with chemotherapy (44). Despite advances in available therapies, there remains an urgent need to further investigate and develop novel therapies, including immunotherapy strategies capable of targeting the minimally immunogenic tumor microenvironment of NB. Consequently, several clinical trials are currently evaluating the antitumor activity of GD2-targeted CAR-T cell therapy in patients with relapsed or refractory NB (NCT03373097, NCT00085930). Early results from these trials demonstrate promising efficacy, particularly in patients with HRNB (45).

In NB, ALK plays a dual role as a therapeutic target and a biomarker of high-risk disease, especially in tumors harboring activating mutations or amplification. Although ALK inhibitors have already entered clinical practice, intrinsic and acquired resistance remain major challenges. Therefore, the presence of full-length ALK on the tumor cell surface and the availability of well-defined ALK-derived epitopes provide a strong rationale for integrating ALK-directed immunotherapies into multimodal treatment, particularly in high-risk ALK-positive NB.

### 3.3 Inflammatory myofibroblastic tumor

Inflammatory myofibroblastic tumor (IMT) is a rare mesenchymal neoplasm composed of spindle-shaped cells and myofibroblasts associated with a variable inflammatory infiltrate composed of plasma cells, lymphocytes, and eosinophils. IMT accounts for less than 1% of all soft tissue tumors and occurs predominantly in children and young adults, although it may arise across a wide age range. IMT can arise in several anatomical sites, including the lungs, abdomen, pelvis and retroperitoneum (46). Despite its generally low or intermediate malignant potential, it may show local invasion, recurrence after resection, and, rarely, metastatic spread (47).

Approximately 50-60% of patients with IMT harbor ALK rearrangements, which lead to the expression of oncogenic ALK fusion proteins and constitutive kinase activation (46). Several fusion partner genes have been identified, such as *TPM3*, *TPM4*, *CLTC*, *CARS*, *ATIC*, SEC31L1, and others, supporting an oncogenic driver role for ALK in a subset of IMT cases (46). As reported in other ALK-rearranged tumors, ALK fusion proteins activate downstream signaling pathways involved in cell proliferation and survival, including MAPK, PI3K/AKT and JAK/STAT signaling.

Surgical resection remains the primary treatment for IMT, particularly for localized disease. In unresectable, recurrent, or metastatic disease, systemic treatment options have historically included anti-inflammatory agents, chemotherapy, and radiotherapy. The identification of recurrent oncogenic kinase fusions has expanded the therapeutic landscape of IMT, supporting the use of molecular treatment strategies in advanced disease. Current guidelines recommend ALK inhibitors as first-line systemic therapy for IMT patients with ALK fusions. TKIs, such as crizotinib, have demonstrated significant efficacy in ALK-positive patients, and second-generation TKIs alectinib and ceritinib have shown activity, even after crizotinib failure (46). The first clinical evidence for crizotinib in IMT came from a 2010 report of an adult patient with ALK-*RANBP2* rearrangement who achieved sustained partial response (48). The FDA approval of crizotinib in 2022 for pediatric patients aged one year and older with unresectable or refractory ALK-positive IMT was supported by the results of the ADVL0912 trial (NCT00939770) (23), together with data from the adult A8081013 trial (NCT01121588). As in other tumors, the emergence of secondary resistance to crizotinib has prompted evaluation of next-generation ALK TKIs. Case reports describe patients with metastatic ALK-positive IMT who achieved renewed clinical responses with ceritinib and later with alectinib after progression on crizotinib, supporting the potential role of sequential ALK inhibition in resistant disease (46, 49). These encouraging observations were reinforced by a multicenter phase I study evaluating ceritinib in children with relapsed or refractory ALK-positive malignancies, including IMT, which showed an ORR of approximately 70% (25). Ongoing studies with brigatinib (Briga-PED trial - NCT04925609) further highlight the growing role of next-generation ALK TKIs in the management of pediatric ALK-rearranged IMT (26, 50).

In ALK-rearranged IMT, ALK TKIs have significantly improved outcomes in unresectable, recurrent, or metastatic disease and are now considered standard systemic therapy. However, given the need for long-term treatment at a young age for many patients, there is a compelling rationale for exploring ALK-directed immunotherapies as a potential strategy to reduce long-term TKI exposure and provide durable disease control.

### 3.4 Rhabdomyosarcoma

Rhabdomyosarcoma (RMS) is the most common soft-tissue sarcoma in children and adolescents and, according to current WHO classification, comprises four main histologic subtypes: embryonal (ERMS), alveolar (ARMS), pleomorphic, and spindle cell/sclerosing RMS (51–53). ARMS is typically more aggressive and is frequently characterized by recurrent chromosomal translocations that generate *PAX3–FOXO1* or *PAX7–FOXO1* fusion genes (**Figure 1B**), whereas ERMS is more genetically heterogeneous, characterized by chromosomal aneuploidy and frequent alterations in RAS signaling pathways (54–56). Despite treatment approaches combining chemotherapy, surgery, and radiotherapy, outcomes remain poor in patients with metastatic, relapsed, or refractory disease (57).

ALK is frequently expressed in RMS, particularly in the alveolar subtype (FP-ARMS), where it has been associated with more aggressive clinical behavior (53). ALK expression correlates with the presence of PAX3/7–FOXO1 fusion, metastatic disease, and adverse clinicopathological features (58). It has been demonstrated that PAX3–FOXO1 fusion protein directly regulates ALK transcription through binding to ALK-associated super-enhancers, resulting in high-level expression of the full-length receptor in FP-ARMS (**Figure 1B**) (59).

However, in contrast to ALCL, neuroblastoma, or IMT, ALK does not appear to function as a dominant oncogenic driver in most RMS cases. Rather, ALK is generally overexpressed at the protein level, while activating mutations, amplifications, or ALK rearrangements are uncommon (60). Notably, these findings are further supported by *in vivo* evidence from a pediatric patient-derived RMS xenograft model, in which treatment with crizotinib failed despite detectable ALK expression (61). Similarly, limited activity was later reported with ceritinib, which achieved only occasional disease stabilization in a small cohort of RMS patients (62). These findings indicate that the presence of ALK protein is not sufficient to confer therapeutic dependence on ALK signaling in RMS. Therefore, in RMS, ALK should be regarded primarily as a potential tumor-associated surface antigen rather than as a validated driver target. Conversely, rare cases harboring ALK genomic alterations, such as ATIC–ALK fusions, achieved a significant and durable tumor reduction with crizotinib after failure of multiple chemotherapy regimens, further supporting the notion that ALK inhibitors may represent a valuable therapeutic option for the small subset of RMS harboring actionable ALK rearrangements (63).

From this perspective, RMS may represent a useful setting for exploring ALK-directed immunotherapies, particularly antibody-based strategies, ADCs, or CAR-T cells, provided that sufficient and homogeneous surface expression can be demonstrated. However, the clinical utility of targeting ALK in RMS remains uncertain and requires careful evaluation, especially given the distinction between simple protein overexpression and true ALK-driven oncogenic addiction.

### 3.5 Other pediatric tumors with ALK expression or rare ALK alterations

#### 3.5.1 Glioblastoma

Glioblastoma (GBM) is a highly aggressive astrocytic tumor and represents the most lethal primary brain malignancy. Rearrangements of ALK have been identified in both astrocytomas and aggressive GBMs. In particular, ALK fusion events such as *LRRFIP1–ALK*, *DCTN1–ALK*, and *PRKD3–ALK*, have been detected in congenital/infant, pediatric, and adult GBMs (64). In contrast, no recurrent ALK mutations have been identified (**Figure 1A**). Beyond these genomic alterations, ALK overexpression has been reported in a subset of GBM cases, and has been associated with the activation of oncogenic signaling pathways involving N-Myc, Sox4, and Akt (64, 65). However, ALK immunohistochemical positivity or overexpression are not indicative of oncogenic ALK dependence, as current evidence does not support a consistent driver role for full-length ALK in most GBMs.

Preclinical studies using patient-derived cells from an infant with ALK-fused congenital GBM demonstrated promising therapeutic activity of ALK inhibitors (65). Notably, a phase Ib clinical trial (NCT02270034) evaluating crizotinib in combination with radiotherapy or temozolomide demonstrated a favorable safety profile and promising efficacy in patients with newly diagnosed GBM (66). Overall, the therapeutic relevance of ALK in GBM is likely restricted to molecularly defined fusion-positive cases, whereas tumors with ALK overexpression alone remain speculative candidates for ALK-directed immunotherapy.

#### 3.5.2 Infant-type hemispheric gliomas

Infant-type hemispheric glioma (IHG) is a high-grade diffuse glioma that arises in the cerebral hemispheres during early childhood. These tumors are characterized by RTK fusions involving the NTRK family, ROS1, ALK, or MET (67). Most cases occur within the first year of life. Histologically, IHGs are highly cellular tumors composed of astrocytic cells with mild-to-moderate nuclear pleomorphism. The oncogenic fusions result in aberrant activation of kinase domains, driving tumorigenesis through the PI3K and/or MAPK signaling pathways. Compared with pediatric diffuse high-grade gliomas arising in older children, IHGs in early childhood are associated with more favorable outcomes, with ALK-rearranged tumors demonstrating the best prognosis among the fusion subtypes (68).

Current treatment includes neurosurgical resection, chemotherapy, radiotherapy, or a combination of these approaches. Recent case reports have demonstrated promising outcomes in ALK-fused IHG patients treated with lorlatinib following partial surgical resection, showing improved objective response rates compared with conventional chemotherapy (69). Moreover, a currently recruiting clinical trial (NCT06333899) is investigating the efficacy of lorlatinib in children newly diagnosed with high-grade glioma harboring ALK or ROS1 fusions to evaluate its safety when administered in combination with chemotherapy or following radiation therapy.

#### 3.5.3 Ewing sarcoma

EwS is a rare, highly malignant small round-cell tumor that represents the second most common primary bone malignancy in children and adolescents. It arises from primordial bone marrow–derived mesenchymal stem cells, but less frequently it originates in soft tissue (70). At diagnosis, metastatic disease is present in up to 25% of patients, and survival is strongly influenced by dissemination. At the molecular level, EwS is characterized by specific chromosomal translocations that fuse *EWSR1* or, less commonly, *FUS* to members of the ETS family of transcription factors. In approximately 85–95% of cases, the most common rearrangement is *EWSR1–FLI1* (t11;22), which is the major oncogenic driver. Less frequently, alternative fusions such as *EWSR1–ERG* occur (**Figure 1B**) (71).

ALK expression has been reported in some patients with EwS, with expression levels varying from low to high (**Figure 1B**) (72). Moreover, reduced ALK expression has been observed in post-chemotherapy specimens compared with primary tumors. Functional studies in Ewing sarcoma cell models further indicate that pharmacological inhibition of ALK signaling can impair tumor cell proliferation, and crizotinib has shown *in vitro* antitumor activity (73, 74). However, no *in vivo* preclinical or clinical studies have yet demonstrated meaningful efficacy of ALK TKIs in EwS (72). Therefore, in this setting ALK is better regarded as a possible tumor-associated antigen than as a validated driver target.

#### 3.5.4 Renal cell carcinoma, thyroid cancer and spitzoid tumors

Renal cell carcinoma (RCC) is a rare cancer in children, accounting for approximately 2–4% of all pediatric renal tumors (75). The clinical presentation of RCC is highly variable, and some patients may remain asymptomatic, often resulting in delayed diagnosis. Recent studies have identified ALK rearrangements in pediatric RCC, defining ALK-rearranged RCC as a distinct emerging subtype of RCC. Several fusion partners have been reported in these tumors, including VCL, TPM3, STRN, EML4, and HOOK1 (76). Notably, STRN-ALK and EML4-ALK fusions have also been detected in pediatric papillary thyroid carcinoma, suggesting overlapping molecular mechanisms across these pediatric cancers (77). ALK rearrangements are present in approximately 10–20% of spitzoid tumors, a group of melanocytic neoplasms characterized by large epithelioid or spindle-shaped cells (78). Although spitzoid tumors predominantly occur in children and adolescents, they may arise at any age.

Overall, ALK functions as a central oncogenic driver in pediatric ALCL, neuroblastoma, and IMT, whereas in other tumors it is more often aberrantly expressed without a clearly established driver role. The partial and sometimes transient benefits of ALK targeted tyrosine kinase inhibitors, together with evidence of ALK immunogenicity, particularly in ALCL, provide a strong rationale for developing and clinically testing ALK directed immunotherapies as complementary strategies to conventional and ALK targeted treatments in pediatric ALK positive tumors.

## 4 ALK as an oncoantigen

ALK has been recognized as an oncoantigen because it combines two key biological properties: it drives malignant transformation and, at the same time, functions as a tumor-associated antigen recognized by the immune system. In many ALK-positive tumors, ALK signaling is critical for tumor cell survival, making it a suitable target for immunotherapy. Moreover, ALK expression in healthy postnatal tissues is very low and highly restricted to a few neurons in the CNS, the spinal cord, and enteric neurons (79). The low background expression and strong oncogenic driver make ALK a particularly important immunotherapy target in ALK-positive tumors and a paradigmatic example of precision oncology in childhood cancer.

Interestingly, both spontaneous humoral and cellular immune responses directed against oncogenic ALK have been identified in patients with ALCL (80). More than 90% of children and adolescents with ALK-positive ALCL exhibit detectable anti-ALK antibodies in serum or plasma (81). Notably, higher antibody titers correlate with improved survival and lower risk of relapse (82). In 2010, a study on 95 patients demonstrated a strong inverse correlation between the strength of the pre-existing anti-ALK antibody response and the risk of relapse after chemotherapy, underscoring the clinical relevance of endogenous anti-ALK immunity (82). In addition, ALK-specific cytotoxic T lymphocytes (CTLs) and CD4+ helper T cells have been detected in peripheral blood mononuclear cells of patients with ALK-positive ALCL, both at diagnosis and during remission, whereas such responses are absent in ALK-negative patients and healthy volunteers (83). These spontaneous T-cell responses are likely initiated by ALK-derived peptides presented on major histocompatibility complex class I (MHC-I) molecules expressed by tumor cells or antigen-presenting cells. Spontaneous immune responses against ALK have also been detected in the peripheral blood of patients with ALK-positive ALCL, including humoral and HLA-A*02:01-restricted CD8+ T cell responses to defined ALK-derived peptides, demonstrating the intrinsic immunogenicity of the oncogenic fusion protein. Tetramer-based studies have detected a measurable repertoire of ALK-specific CD8+ T cells in healthy donors. Although healthy donors and patients exhibited comparable numbers of CD8+ anti-ALK T cells, their functional phenotypes differed. In patients, the anti-ALK CD8+ T-cell repertoire contained a substantial proportion of effector and memory T cells, whereas that from healthy donors exhibited a naïve phenotype. High frequencies of CD8+ ALK-specific cells, together with the presence of a long-lasting ALK-specific memory T-cell population, support the notion that ALK can function as a tumor-associated antigen that elicits adaptive immune responses (84). In addition, CD4+ T-cell responses against NPM-ALK have been identified in patients with ALCL in remission. ALK-specific CD4+ T cells were detected following stimulation with autologous dendritic cells pulsed with long overlapping ALK peptide pools. Notably, the identified CD4+ T-cell epitopes were predominantly located within the ALK-derived portion of the fusion protein. Furthermore, Stadler et al. identified an immunogenic neoepitope spanning the NPM-ALK fusion junction and its corresponding HLA-DR13-restricted T-cell receptor (TCR) (83). Consequently, therapeutic approaches aimed at enhancing anti-ALK immunity, including cancer vaccines and adoptive cellular immunotherapies, may provide significant clinical benefit in most ALK+ tumors.

**Figure 2:**
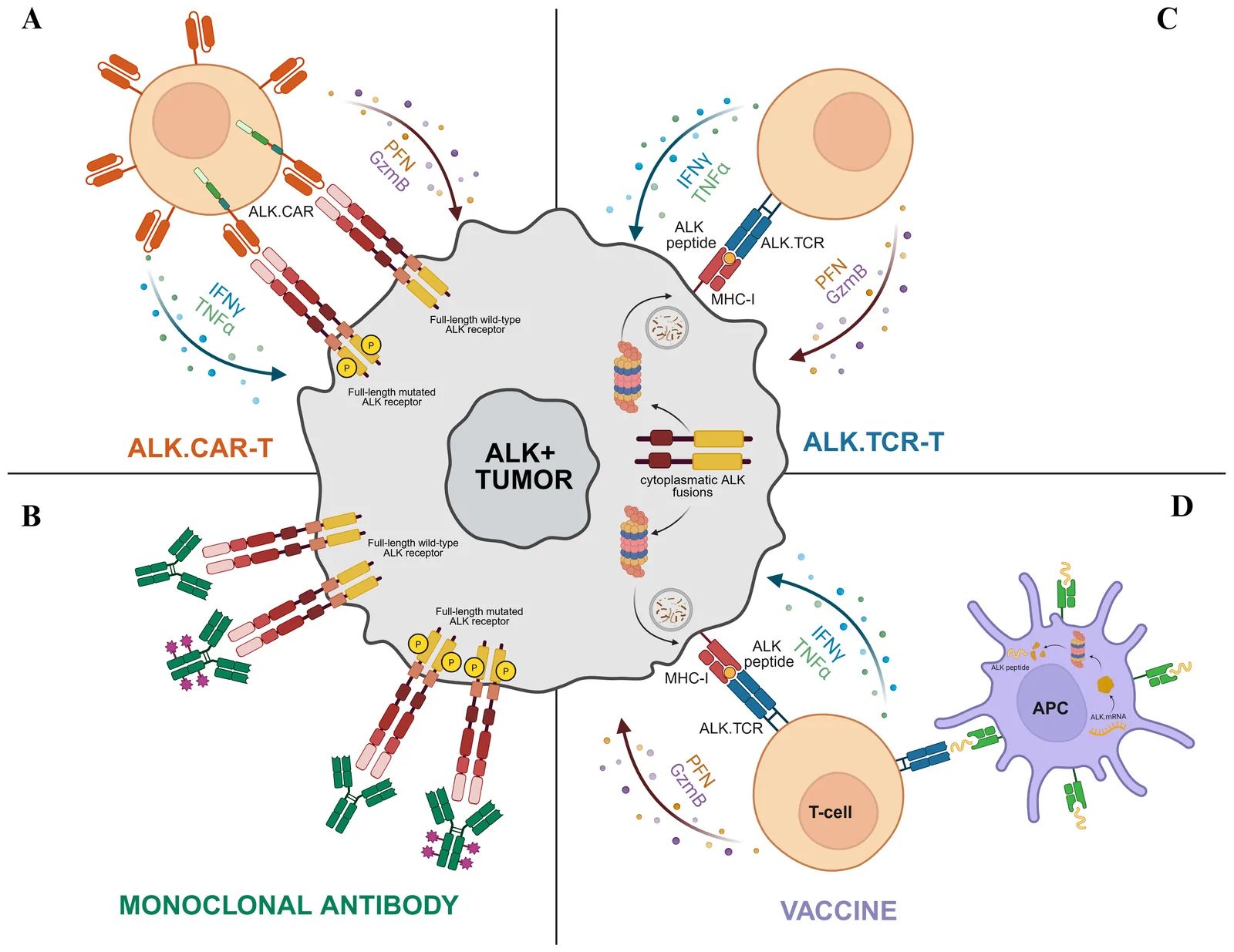
Immunotherapeutic strategies targeting ALK-positive tumors. Tumor cells expressing transmembrane full-length wild-type, mutated, or amplified ALK can be targeted by anti-ALK monoclonal antibodies, antibody–drug conjugates (ADCs), or chimeric antigen receptor (CAR) T cells engineered to recognize ALK. **(A)** ALK-CAR-T cells are genetically engineered T cells expressing CARs specific for ALK on the tumor cell surface. **(B)** Anti-ALK antibodies and ALK-directed ADCs. Anti-ALK antibodies block ALK ligand-binding and inhibit oncogenic signaling, whereas ADCs combine receptor blockade with intracellular delivery of cytotoxic agents, resulting in tumor cell killing. Tumor cells harboring ALK cytoplasmic fusion proteins present ALK-derived peptides through major histocompatibility complex class I (MHC-I) or MHC-II. **(C)** ALK-TCR-T cells are engineered to express T cell receptors (TCRs) recognizing ALK peptides presented by MHC-I or MHC-II molecules. **(D)** ALK vaccines, including peptide-based or mRNA-based platforms, deliver entire portion of ALK or selected ALK peptides to antigen-presenting cells (APCs), thereby promoting priming and expansion of ALK-specific CD8+ T cells. ALK-CAR-T, ALK-TCR-T, and vaccine-induced ALK-specific T cells mediate anti-tumor activity through secretion of inflammatory cytokines, including IL-2, interferon-γ (IFNγ), and tumor necrosis factor (TNF), as well as cytolytic effector mechanisms involving perforin (PFN) and granzyme B (GZMB).

In ALK-driven tumors, immune escape through complete loss of ALK expression may be less likely than for lineage-associated antigens such as CD30 or GD2, because ALK is directly implicated in oncogenic signaling through aberrant expression or constitutive activation, resulting in a stronger oncogenic dependency. This dependency may limit the emergence of fully antigen-negative clones. Nevertheless, this does not preclude other mechanisms of immune evasion, such as reduced surface density, impaired trafficking or internalization, that may limit the efficacy of surface-targeting immunotherapies despite persistent ALK dependency. Overall, these observations provide a strong rationale for developing ALK-directed immunotherapies (**Figure 2**).

## 5 ALK-directed immunotherapies

ALK alterations are important oncogenic drivers in several pediatric cancers. Although targeted therapies against ALK have demonstrated promising clinical activity, treatment resistance and disease relapse remain significant challenges. Importantly, these limitations have increased interest in the application of ALK-directed immunotherapies for pediatric malignancies, both as monotherapies and in combination with ALK inhibitors to improve the durability of responses and long-term clinical outcomes. In this context, it is important to distinguish among the different biological settings in which ALK is detected in pediatric tumors, including ALK fusions, activating point mutations, gene amplification, and mere protein overexpression. Whereas ALK fusions, activating mutations, and high-level amplification generally support ALK-driven oncogenic signaling and represent validated settings for ALK-targeted therapy, tumors characterized only by ALK protein overexpression or IHC positivity should be considered more cautiously, as they are likely to be less dependent on ALK and therefore more speculative candidates for ALK-directed immunotherapy, particularly when antigen density is low.

### 5.1 ALK vaccines

Evidence that ALK is naturally immunogenic and elicits spontaneous humoral and cellular immune responses in patients with ALK+ tumors prompted the development of ALK-specific vaccination strategies (85). The first direct demonstration that vaccination against ALK could confer protective antitumor immunity was obtained using DNA-based vaccine platforms in murine ALCL models. Immunization with plasmids encoding portions of the cytoplasmic domain of ALK was shown to protect mice from both local and systemic lymphoma growth and was associated with ALK-specific IFN-γ secretion and CD8+ T cell-mediated cytotoxicity. Combined administration of chemotherapy and DNA vaccination significantly extended survival in mice challenged with ALK-positive lymphomas (86).

This vaccine was subsequently applied to a transgenic murine model of ALK-rearranged NSCLC, where it elicited tumor-specific cytotoxic responses and prevented tumor growth. In this setting, efficacy was improved by co-administration of ALK TKIs, and in tumor models with high PD-L1 expression, addition of anti-PD-1 antibody was required to overcome vaccine resistance (87). Peptide-based vaccine platforms have produced compelling preclinical results in ALK-rearranged NSCLC, with mechanistic insights directly relevant to pediatric tumors. The poor responsiveness of ALK-rearranged tumors to immune checkpoint inhibitors was found to stem from insufficient CD8+ T cell priming against ALK antigens, rather than from intrinsic resistance at the effector phase. A single peptide vaccination was sufficient to restore ALK-specific CD8+ T cell priming, and, in combination with ALK TKIs, led to eradication of established lung tumors and prevention of brain metastasis in preclinical models (88).

Moreover, immunogenic ALK peptides presented by HLA-A*02:01 and HLA-B*07:02, were identified and shown to be recognized by CD8+ T cells from patients with ALK-positive NSCLC, establishing the immunological groundwork for clinical translation (88). Although these studies were performed in ALK-positive NSCLC, an adult-type tumor model, the identification of immunogenic ALK peptides restricted by HLA-A*02:01 and HLA-B*07:02 and their recognition by patient-derived CD8+ T cells provides a strong rationale for translating peptide-based vaccination to ALK-driven pediatric tumors in HLA-matched population.

Recently, an mRNA-based vaccine encoding the cytoplasmic domain of ALK formulated in lipid nanoparticles using Moderna technology was developed. ALK-mRNA vaccination induced robust ALK-specific CD8+ and CD4+ T-cell responses directed against immunogenic regions of ALK. In a preventive setting, this vaccine delayed tumor growth and prolonged survival in a low MHC-I ALK-positive syngeneic lung tumor model (89).

Overall, these findings support further investigation of the ALK-mRNA vaccine in therapeutic settings for ALK-positive tumors and provide a rationale for early-phase clinical trials to investigate its safety, optimal dosing and efficacy in humans. In the pediatric setting, ALK vaccines represent a rational immunotherapeutic approach for ALK-associated tumors by targeting recurrent activating mutations in high-risk neuroblastoma and ALK fusion proteins in ALCL and IMT. Fusion junction–derived neoantigens offer highly tumor-specific targets, as they are absent from normal tissues, thereby supporting both specificity and safety in ALK-directed vaccination strategies, especially during the minimal residual disease. However, key challenges remain for clinical translation, including designing multiepitope vaccines to cover diverse HLA backgrounds and assessing the risk of autoimmunity in tissues expressing low levels of ALK.

### 5.2 ALK antibodies

For tumors that express the full-length receptor, such as NB, FP-ARMS, and IMT, ALK antibodies may be a therapeutic option, either as blocking agents or as antibody–drug conjugates (ADCs). In 2005, a study conducted in human cell lines demonstrated that ALK-targeting antibodies were able to block ALK activity and inhibit downstream signaling pathways (90). Furthermore, Carpenter et al. showed that antagonistic ALK antibodies inhibited cell proliferation and induced antibody-dependent cellular cytotoxicity *in vitro* in neuroblastoma-derived cell lines expressing either wild-type or mutant ALK (91). Additionally, treatment with crizotinib enhanced the sensitivity of NB cells to antibody-mediated growth inhibition by promoting the accumulation of ALK on the cell surface, thereby increasing antigen accessibility for antibody binding (91).

ADCs represent a rapidly expanding class of anticancer therapeutics that combine the selective targeting capabilities of monoclonal antibodies (mAbs) with the potent cytotoxic activity of anticancer agents. The development of the chimeric ALK-targeting ADC CDX-0125-TEI demonstrated selective activity against both wild-type and mutant ALK neuroblastoma cells. CDX-0125-TEI showed efficient antigen binding, rapid internalization, and strong cytotoxic effects at picomolar concentrations across cells with varying levels of surface ALK expression. *In vivo* studies using patient-derived neuroblastoma xenograft models confirmed the therapeutic efficacy of CDX-0125-TEI, highlighting the potential of ALK-directed ADCs as a promising treatment strategy for NB (92).

Recently, Guerra et al. demonstrated that CDX0239-PBD, a humanized ALK-targeting antibody conjugated to pyrrolobenzodiazepine (PBD), is efficiently internalized in ALK-expressing NB cell lines and induces ALK-dependent cell-surface cytotoxicity. CDX0239-PBD showed potent antitumor activity, achieving complete responses in cell line-derived xenograft models of NB and FP-ARMS that express high levels of ALK on the cell surface. These findings support the clinical development of ALK-directed ADCs for the treatment of multiple pediatric and adult ALK-expressing malignancies (93). Characterization of ALK antibody binding sites has provided important mechanistic insights into the biological activity of ALK-targeting antibodies and may facilitate the rational design of next-generation antibodies with enhanced therapeutic efficacy (94). Additionally, structural studies of the interactions between ALK and its ligands, ALKAL1 and ALKAL2, may enable the development of highly specific antibodies capable of effectively blocking ALK signaling in tumors such as NB (95). Despite this encouraging progress, there are no current clinical trials with ALK-directed ADC.

### 5.3 ALK-directed T-cell engagers

T-cell engagers (TCEs) have emerged as a promising class of cancer immunotherapies and are increasingly being evaluated for the treatment of both hematological and solid tumors. TCEs are engineered fusion proteins that combine a CD3-binding antibody with a tumor antigen-specific antibody, thereby redirecting and activating T cells to selectively eliminate tumor cells (96). Several TCEs have already received FDA approval, while others are currently undergoing preclinical and clinical evaluation (97). In 2024, Chuan Chen et al. reported the identification of a fully human antibody variable heavy (VH) domain, designated VH20, which binds ALK with high specificity and affinity. Subsequently, VH20-OKT3-7-scFv TCE was developed by linearly fusing VH20 to the humanized anti-human CD3 single-chain variable fragment, OKT3-7-scFv. Moreover, a human IgG1 Fc domain was fused to the TCE to improve stability and binding activity (98). *In vitro* studies demonstrated specific cytotoxic activity against ALK-expressing tumor cells, including a panel of neuroblastoma cell lines harboring either wild-type or mutant ALK. In particular, the VH20-TCE efficiently mediated tumor cell killing by engaging both resting and pre-activated T cells (98). Despite these promising *in vitro* findings, no preclinical *in vivo* studies have yet been reported to evaluate the antitumor efficacy, pharmacokinetics, or safety profile of using TCE for the treatment of ALK-positive tumors.

### 5.4 T cell-based approaches

#### 5.4.1 ALK.CAR-T

CAR-T cell therapy targeting ALK has emerged as a rational therapeutic strategy for ALK-positive solid tumors, particularly NB. The rationale for this approach is the expression of full-length ALK at the tumor cell surface, where the receptor functions as both a central oncogenic driver and a tumor-associated antigen accessible to CAR-T cell targeting (99). Full-length ALK is expressed across multiple NB cell lines and primary tumor specimens, although with substantial intertumoral variability at both transcript and protein levels (100, 101). ALK expression differs across molecular and clinical subgroups of NB, including MYCN-defined contexts, and may also carry prognostic significance (102). This heterogeneous pattern of surface expression is likely to have important therapeutic implications, as variability in antigen density may directly influence the efficacy of ALK-directed immunotherapeutic strategies. Beyond differences in expression levels, ALK surface expression may also be influenced by the biological properties of oncogenic ALK variants. Activating mutations such as F1174 and R1275 have been shown to impair physiological receptor trafficking by constitutively activating the kinase, thereby altering receptor localization and membrane distribution (103). These observations suggest that antigen accessibility in ALK-driven tumors is regulated not only at the transcriptional level but also by post-translational mechanisms governing receptor trafficking and turnover.

Walker et al. developed one of the first ALK-directed CAR-T cell platforms and demonstrated its preclinical antitumor activity in ALK-expressing tumor models. Their study showed that ALK.CAR-T cells selectively recognized and eliminated ALK-positive tumor cells while largely sparing normal tissues with physiologically low antigen expression. Notably, CAR-T functionality was highly dependent on antigen density, with increased ALK surface expression correlating with enhanced cytokine production, proliferation, and *in vivo* antitumor efficacy (104).

Bergaggio et al. further strengthened the rationale for ALK.CAR-T cell therapy. Using a different ALK.CAR construct, they demonstrated that modulation of ALK expression can significantly enhance CAR-T cell activity against neuroblastoma models. In high-ALK neuroblastoma models, ALK.CAR-T cells achieved tumor eradication comparable to GD2.CAR-T cells. In contrast, tumors with low surface ALK expression proved largely refractory to ALK.CAR-T monotherapy, identifying antigen density as a principal determinant of therapeutic response. Mechanistically, treatment with ALK TKIs, such as lorlatinib, was shown to upregulate surface ALK expression through reduced receptor internalization and transcriptional de-repression, selectively enhancing ALK.CAR-T cell activity without affecting GD2.CAR-T cells (99).

These findings provide a mechanistic basis for combinatorial strategies that integrate ALK TKIs with adoptive CAR-T cell immunotherapy and have direct implications for patient stratification and treatment scheduling in ALK-driven neuroblastoma. These preclinical insights have provided the foundation for early-phase clinical evaluation of ALK-targeted CAR-T approaches (NCT06803875). Conceptually, ALK-directed CAR T cells could be applied to other pediatric tumors that express full-length ALK receptor at the cell surface, such as subsets of RMS, IMT, and EwS. However, in these settings, where ALK is typically expressed at a lower density, ALK.CAR-T therapy will mostly be combined with agents that increase antigen expression or ALK.CAR-T cells will be designed to potentiate their killing capacity.

#### 5.4.2 ALK.TCR-T

Tumors harboring ALK fusions that are expressed intracellularly and lack ALK on the surface are not amenable to conventional CAR T-cell targeting. In these settings, TCR-engineered T cells (TCR-T) that recognize intracellular proteins presented as peptide–HLA complexes represent a more suitable therapeutic strategy. Consequently, TCR-based therapies may be developed for ALK-driven tumors by engineering T cells with receptors specific for ALK-derived peptides presented by defined HLA molecules.

Initial studies demonstrated the feasibility of cloning TCRs that recognize ALK-derived peptides presented by HLA-B*07:02 from mice vaccinated with human ALK peptides (105). These findings provide the basis for the development of two engineered CD8+ ALK-specific TCR-Ts, which exhibited potent and selective *in vitro* anti-tumor activity across multiple ALK-positive ALCL cell lines expressing HLA-B*07:02 (106). In subsequent *in vivo* experiments, a first generation of ALK-specific TCR-T cells demonstrated durable anti-tumor responses in an HLA-B*07:02 expressing metastatic ALK-positive NB model, without evidence of on-target/off-tumor or off-target toxicity (107).

Altogether, these data demonstrate that ALK-specific TCR-T cells can achieve high specificity and anti-tumor efficacy across multiple ALK-driven tumor models and support their development as a therapeutic strategy for tumors harboring ALK fusions. Additional support for this approach comes from recent studies that identified CD4+ TCRs in patients with ALK-positive ALCL that specifically recognize peptides derived from the ALK portion of the fusion protein. Importantly, one of these CD4+ TCRs was shown to recognize an NPM-ALK fusion neoepitope presented by a human MHC class II complex, further highlighting the immunogenic potential of ALK fusion-derived neoantigens (83).

## 6 Barriers to translation

Despite the considerable therapeutic promise of ALK-targeted immunotherapies, several translational challenges remain before these approaches can be broadly implemented in pediatric oncology. To note, these barriers differ across CAR-T cells, TCR-T cells, and peptide vaccines, reflecting the distinction between tumors that express ALK on the cell surface and those driven by intracellular ALK fusion proteins.

First, although ALK expression is largely restricted to subsets of neurons within the central nervous system, spinal cord, and enteric nervous system, this physiological expression raises important safety concerns for highly potent immune-based therapies, including CAR-T cells, TCR-T cells, and therapeutic vaccines. While preclinical studies in murine models have suggested an acceptable safety profile, these findings cannot fully predict human neurotoxicity because murine ALK expression patterns, blood-brain barrier physiology, and major histocompatibility complex biology differ from those of humans. Experience with other immune-targeted therapies has demonstrated that even low-level antigen expression in normal tissues can lead to severe on-target/off-tumor toxicity, highlighting the importance of careful neurological assessment, long-term surveillance, and cerebrospinal fluid monitoring (108). One strategy to mitigate potential toxicity is the incorporation of safety mechanisms, such as suicide switches. Wei Xiao et al. demonstrated that the addition of the inducible caspase-9 (iCasp9) suicide gene system to FGFR4. CAR-T cells in RMS contest could enable rapid elimination of CAR-T cells in the event of significant toxicity upon administration of AP20187 (109). Similarly, iCasp9-engineered GD2 CAR-T cells have shown a safety profile with limited toxicities and modest efficacy in NB (110, 111). These approaches support the potential role of safety switches in improving the tolerability and clinical applicability of CAR-T cell therapies for pediatric tumors.

Second, the highly immunosuppressive tumor microenvironment of solid tumors, particularly NB, presents a major obstacle to effective immunotherapy. NB is considered a cold tumor and is characterized by low T-cell infiltration, impaired antigen presentation, abundant tumor-associated macrophages and myeloid-derived suppressor cells, regulatory T cells, and expression of inhibitory immune checkpoints. These features can substantially reduce the efficacy and persistence of CAR-T cells, TCR-T cells, and vaccine-induced immune responses (112, 113). Consequently, combination strategies that remodel the tumor microenvironment, including checkpoint blockade, cytokine modulation, macrophage reprogramming, or stromal targeting, will likely be required to achieve durable clinical responses (112). Recently, Strijker et al. identified 13 immunosuppressive interactions within the NB TME through single-cell RNA sequencing (scRNA-seq), revealing a pivotal role for Macrophage Migration Inhibitory Factor (MIF) in suppressing CAR-T cell efficacy. To translate these findings into a clinically applicable therapeutic strategy, researchers employed PROTAC technology to target MIF, resulting in significantly enhanced activation and antitumor activity of CAR-T cells directed against GPC2 and B7-H3 in NB (114).

Finally, TCR-based therapies and peptide vaccines remain constrained by HLA restriction, limiting patient eligibility within the genetically diverse pediatric population. Although several immunogenic ALK-derived epitopes have been identified, most are presented by relatively common HLA alleles such as HLA-A*02:01, excluding a substantial proportion of children worldwide. In addition, individualized HLA typing, epitope selection, and autologous T-cell manufacturing increase treatment complexity, cost, and production time. Expanding the repertoire of validated ALK epitopes across multiple HLA haplotypes and developing scalable manufacturing strategies will therefore be essential to improve the feasibility and equitable clinical implementation of ALK-directed cellular and vaccine immunotherapies. Emerging mRNA vaccine platforms may partially bypass these constraints by enabling multiepitope or polyantigenic constructs that can be adapted to diverse HLA backgrounds, potentially broadening patient eligibility while preserving antigen specificity.

## 7 Conclusions and future perspectives

The discovery of ALK alterations across a broad spectrum of pediatric malignancies and the clinical success of ALK TKIs have significantly established ALK as one of the most relevant targets in childhood cancer (4). At the same time, the restricted postnatal expression of ALK and its intrinsic ability to elicit spontaneous humoral and cellular immune responses support its designation as an oncoantigen and provide a strong biological rationale for ALK-directed immunotherapies (81). Although ALK TKIs have improved outcomes in ALK-driven malignancies such as NB, ALCL, and IMT, their long-term efficacy is limited by acquired resistance and persistent MRD. These limitations highlight the need for immune-based strategies that can provide long-term tumor control.

In this context, ALK-directed immunotherapies are emerging as means to address these unmet needs. Monoclonal antibodies, antibody–drug conjugates, CAR-T cells, TCR-T cells, and vaccines have shown antitumor activity and robust ALK-specific immune responses in preclinical models (86, 89, 92, 93, 99, 106, 107). While current evidence remains largely preclinical and no ALK-directed immunotherapy has yet demonstrated clinical benefit in pediatric patients, these data justify early-phase clinical development, in combination with either ALK-TKIs or conventional therapies.

In principle, ALK-targeted vaccines could enhance the priming, expansion, and persistence of endogenous ALK-specific T cells, whereas ALK-specific TCR-T and CAR-T cells could provide immediate and potent antitumor effector activity. The integration of these approaches has the potential to generate complementary or synergistic antitumor effects, simultaneously engaging endogenous and engineered ALK-specific T-cell populations to eradicate residual persister cells and reduce the risk of relapse. In conclusion, ALK represents a unique intersection between precision oncology and cancer immunotherapy. Although the integration of multiple ALK-directed immunotherapeutic strategies remains a future perspective rather than an established therapeutic paradigm, it warrants systematic evaluation as a potential next-generation approach for children with ALK-positive malignancies.

## Glossary

ADC: Antibody–drug conjugate
AKT: Protein Kinase B
ALCL: Anaplastic large cell lymphoma
ALK: Anaplastic lymphoma kinase
ALKAL1: ALK and LTK ligand 1
ALKAL2: ALK and LTK ligand 2
ARMS: Alveolar Rhabdomyosarcoma
ATIC: 5-Aminoimidazole-4-Carboxamide Ribonucleotide Formyltransferase/IMP Cyclohydrolase
B7-H3: B7 homolog 3 protein
BV: Brentuximab-vedotin
CARS: Cysteinyl-tRNA Synthetase
CAR-T: Chimeric antigen receptor T cells
CDK4: Cyclin-dependent kinase 4
CLTC: Clathrin Heavy Chain
CNS: Central nervous system
CR: Complete Response
CTLs: Cytotoxic T lymphocytes
DCTN1: Dynactin Subunit 1
EML4: Echinoderm Microtubule-Associated Protein-Like 4
ERG: ETS-related gene
ERK: Extracellular signal-regulated kinase
ERMS: Embryonal Rhabdomyosarcoma
ETS: Erythroblast transformation-specific
EwS: Ewing sarcoma
EWSR1: Ewing sarcoma breakpoint region 1 gene
FGFR4: Fibroblast growth factor receptor 4
FOXO1: Forkhead box protein O1
FP-ARMS: Fusion positive Alveolar Rhabdomyosarcoma
FUS: FUS gene
GBM: Glioblastoma
GD2: Disialoganglioside
GPC2: Glypican 2
GZMB: Granzyme B
HLA-A: Human Leukocyte Antigen Locus A
HOOK1: Hook microtubule tethering protein 1
HRNB: High-risk neuroblastoma
IFN-γ: Interferon gamma
IHG: Infant-type hemispheric glioma
IMT: Inflammatory myofibroblastic tumors
JAK: Janus kinase
LDL: Low Density Lipoprotein
LRRFIP1: Leucine Rich Repeat In FLII Interacting Protein 1
MAM: Meprin, A-5 protein, and receptor protein-tyrosine phosphatase mu
MAPK: Mitogen-Activated Protein Kinase
MDD: Minimal disseminated disease
MDM2: Mouse double minute 2 homolog
MET: Mesenchymal-Epithelial Transition
MHC-I: Major histocompatibility complex class I
MIF: Macrophage Migration Inhibitory Factor
MMAE: Antimitotic agent monomethyl auristatin E
MRD: Minimal residual disease
mTOR: Mammalian Target of Rapamycin
MYCN: MYCN Proto-Oncogene, BHLH Transcription Factor
NB: Neuroblastoma
NHL: Non-Hodgkin lymphomas
NPM1: Nucleophosmin
NSCLC: Non Small Cell Lung Cancer
NTRK: Neurotrophic Tyrosine Receptor Kinase
OKT3: muromonab-CD3
ORR: Overall Response Rate
PAX3: paired box gene 3
PBD: Pyrrolobenzodiazepine
PD-L1: Programmed Death-Ligand 1
PFN: Perforin
PHOX2B: Transcription factor paired mesoderm homeobox protein 2B
PI3K: Phosphatidylinositol 3-kinase
PRKD3: Protein Kinase D3
PROTAC: Proteolysis Targeting Chimera
RANBP2: RAN binding protein 2
RAS: Rat sarcoma
RCC: Renal cell carcinoma
RMS: Rhabdomyosarcoma
ROS1: Proto-oncogene tyrosine-protein kinase 1
RTK: Receptor tyrosine kinase
SEC31L1: Exocyst complex component 1 like 1
Sox4: SRY-box transcription factor 4
STAT3: Signal transducer and activator of transcription 3
STAT5: Signal transducer and activator of transcription 5
STRN: Striatin
TCE: T-cell engager
TCR: T cell receptor
TCR-T: TCR-engineered T cells
TKI: Tyrosine Kinase ihibitor
TNF: Tumor necrosis factor
TPM3: Tropomyosin 3
TPM4: Tropomyosin 4
VCL: Vinculin.
